# The Development of Infants’ Sensitivity to Behavioral Intentions when Inferring Others’ Social Preferences

**DOI:** 10.1371/journal.pone.0135588

**Published:** 2015-09-18

**Authors:** Young-eun Lee, Jung-eun Ellie Yun, Eun Young Kim, Hyun-joo Song

**Affiliations:** 1 Department of Psychology, Yonsei University, Seoul, Korea; 2 Graduate School of Education and Information Studies, University of California, Los Angeles, Los Angeles, United States of America; 3 Department of Occupational Therapy, College of Medical Science, Soonchunhyang University, Asan, Korea; Birkbeck, University of London, UNITED KINGDOM

## Abstract

The present study investigated whether infants reason about others’ social preferences based on the intentions of others’ interactive actions. In Experiment 1, 12-month-old infants were familiarized with an event in which an agent either successfully helped a circle to climb up a hill (successful-helping condition) or failed to help the circle to achieve its goal (failed-helping condition). During the test, the infants saw the circle approach either the helper (approach-helper event) or the hinderer (approach-hinderer event). In the successful-helping condition, the 12-month-old infants looked for longer at the approach-hinderer event than at the approach-helper event, but in the failed-helping condition, looking times were about equal for the two test events. These results suggest that 12-month-old infants could not infer the circle’s preference when the helper’s action did not lead to its intended outcome. In Experiment 2, 16-month-olds were tested in the failed-helping condition; they looked longer at the approach-hinderer event than at the approach-helper event, which suggests that they could reason about the third party’s social preferences based on the exhibited intentions. In Experiment 3, 12-month-olds were familiarized with events in which the final outcomes of helping and hindering actions were ambiguous. The results revealed that 12-month-old infants are also sensitive to intentions when inferring other’s social preferences. The results suggest that by 12-months of age, infants expect an agent to prefer and approach another who intends to help the circle to achieve its goal, regardless of the outcome. The current research has implications for moral reasoning and social evaluation in infancy.

## Introduction

As adults, we readily reason about others’ preferences when watching social interactions. Consider the following situation: while a boy named John is struggling to build a tower of blocks, his friend Ben tries to help him but tears the blocks down by mistake. In contrast, another friend, Mary, destroys the blocks on purpose to tease John. You can easily infer that John would prefer Ben to Mary and would invite Ben, not Mary, to play with him again later. Thus, the intentions behind Ben’s and Mary’s behaviors are critical in determining John’s preference even though the outcome is identical (the tower of blocks has been demolished). As in this example, when understanding why others choose to approach or avoid a particular person, we typically reason about their actions in terms of preference, often relying on the intentions that they exhibited during previous interactions. Such information can help us predict and interpret others’ future actions. The current research examined whether infants also consider intentionality when inferring others’ preferences.

When attributing an agent’s disposition toward another agent, infants attend to the previous history of social interactions between the agents [1–4]. For example, in one experiment [2], 10-month-old infants were habituated to an event in which an agent (either a triangle or a square) helped a circle reach the top of a hill by pushing the circle upward. Next, they were habituated to an event in which the circle was hindered from reaching the top of the hill by another agent that pushed it down the hill. After the habituation phase, the infants were presented with test events in which the circle moved toward and settled next to the helper or the hinderer. The infants looked longer at an event in which the circle approached a hinderer than they did at an event in which it approached a helpful agent. This, and other previous findings, suggests that 9- to 12-month-old infants distinguish helping from hindering actions [2–4]. In addition, by 12-months of age, infants can generate not only retrospective evaluations, but also online predictions of a third party’s social preference on the basis of their previous interactions [1].

Note that the results of the previous studies described above do not provide clear evidence as to whether infants simply attend to behavioral outcomes (positive vs. negative) or reason about the underlying intentions to help or hinder. More specifically, in most of the previous studies, an agent’s helping action was always followed by successful achievement of the recipient’s goal, and an agent’s hindering action was always followed by failure to achieve the goal.

Hamlin [5] recently examined whether infants become sensitive to intentions during moral evaluations in their first year of life. For example, 5- and 8-month-old infants were presented with events in which a character attempted but failed to help the protagonist (failed helper) and the other character attempted but failed to hinder the protagonist (failed hinderer). After watching such events, 8-month-old, but not 5-month-old, infants were more likely to reach for the failed helper over the failed hinderer. Thus, by the age of 8 months, infants develop their own preference toward those with positive behavioral intentions over those with negative behavioral intentions, regardless of the behavioral consequences [5]. This evidence shows that infants can privilege intention over outcome in moral evaluation at an age much younger than has been suggested by previous studies (e.g., [6–8]).

However, previous research has not addressed the question of whether infants can infer others’ preferences toward other agents on the basis of the intentions that motivated their previous interactions. The current research examined this issue by measuring infants’ looking times during events in which an agent approached another who had previously intended to help the agent.

Our task is a more stringent test of infants’ sensitivity to the intentions behind others’ morally relevant behaviors than the choice task used by Hamlin [5], because reasoning about others’ preferences is more cognitively demanding than deciding one’s own preference. Unlike in a choice task, in which infants simply determine their own attitude toward another agent, infants have to represent the internal states of a direct recipient of helping or hindering behaviors when reasoning about an agent’s predilection toward the helper or hinderer. Previous research has demonstrated this. Some earlier evidence suggests that inferring about a third party’s social preference may be more difficult than determining one’s own preferences toward agents. Hamlin et al. [2] measured 6- and 10-month-old infants’ own reach toward the helper or hinderer, as well as their looking times for each test event. In the choice task, both the 6- and 10-month-olds were more likely to reach for the helper than for the hinderer, suggesting that by 6-months-old, infants develop their own preferences based on their evaluation of helpful and hindering individuals. During the looking-time task, the 10-month-olds looked for longer at the approach-hinderer event than at the approach-helper event. However, the 6-month-old infants looked at both events for an equal length of time. Both the 6- and 10-month-olds developed their own preferences for the helper, but only the 10-month-old infants were able to infer a third-party’s preference for another agent.

Using the violation of expectation paradigm, we built on the previous studies by Kuhlmeier, Hamlin, and colleagues to examine whether infants reason about an agent’s social preference toward another agent based on intentions exhibited in past interactions. In Experiment 1, 12-month-olds were assigned to either a successful-helping or a failed-helping condition. We tested 12-month-olds because some previous studies have demonstrated that infants around this age accurately attribute a disposition to an agent based on past social interactions in experiments using similar stimuli (e.g., [1, 4]). In the successful-helping condition, we used computer-animated events similar to those used by Kuhlmeier et al. [4]. The infants were first familiarized with two kinds of events. In the helping event, a circle attempted to climb a hill and another agent (either a square or a triangle) helped the circle reach the top of the hill. In the hindering event, the other agent hindered the circle from climbing the hill. In the failed-helping condition, the infants watched similar familiarization events with the difference that in the helping event, the agent who attempted to help the circle failed to do so; therefore, at the end, the two agents rolled down the hill together. Thus, the outcomes of the helping and hindering events were the same in the failed-helping condition. During the test, the infants in both groups watched events in which the circle approached either the helper (approach-helper event) or the hinderer (approach-hinderer event). We predicted that, if infants expected that the circle would approach the agent who intended to help regardless of the outcome, the infants in both conditions would look for significantly longer at the approach-hinderer event than at the approach-helper event.

## Experiment 1

### Methods

#### Participants

Thirty-two healthy, full-term infants participated in the study (17 female, mean age = 12 months and 18 days, age range = 11 months and 10 days to 13 months and 28 days). Another 21 infants were tested but not included in the analyses because of fussiness (6 in the successful-helping condition, 4 in the failed-helping condition), inattentiveness (5 in the successful-helping condition, 3 in the failed-helping condition), parental interference (2), or drowsiness (1). Half of the infants were randomly assigned to the *successful-helping* condition, and the other half to the *failed-helping* condition. Within each condition, 8 infants observed the approach-helper event, and the other 8 infants observed the approach-hinderer event.

The infants in this and the subsequent experiments were recruited through the posting of recruitment advertisements on online parenting communities and the distribution of leaflets introducing infant development research at a public health center. The infants received a book in return for their participation, and their parents were offered reimbursement for their transportation expenses. Each infant’s parent gave written informed consent, and the protocol was approved by the Institutional Review Board at Yonsei University.

#### Stimuli and procedure

During the experiment, the infant sat on a parent’s lap centered in front of a 22-inch LCD monitor (LG L226WTQ); the infant’s head was approximately 62 cm from the monitor. Parents were instructed to close their eyes and to remain silent and neutral during the entire experiment.

Infants underwent 6 familiarization trials, 1 pre-test display trial, and 1 test trial. During each trial, the infants watched computer-animated videos that were adopted from Kuhlmeier et al. [4]. The videos showed the movements of three agents with eyes and a nose―a red circle, a yellow square, and a green triangle―along two blue hills against a white background.

**Fig 1:**
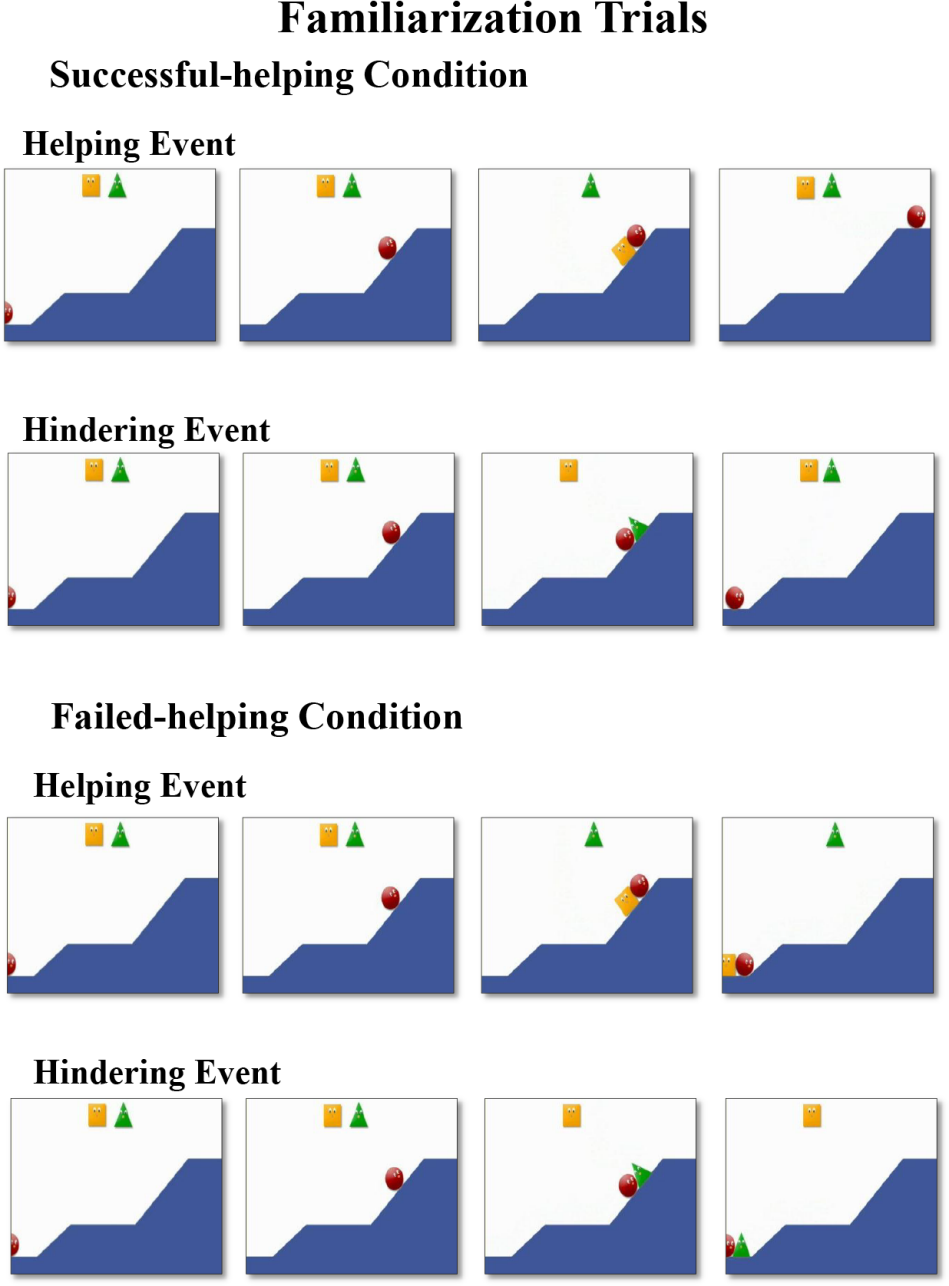
Example of familiarization trials in the successful-helping and the failed-helping conditions.

At the beginning of the familiarization trials in the successful-helping condition, a red circle was located at the bottom of the first of the two hills, and a triangle and a square were in the middle of the upper part of the screen (see Fig 1). Then, the circle reached the top of the first hill and then attempted to reach the top of the second hill but slid back to the base of the second hill. On its second attempt to climb the second hill, the circle was either pushed to the top by the helper (e.g., a square; helping event), or pushed to the bottom by the hinderer (e.g., a triangle; hindering event). At the end of the events, the helper and the hinderer moved back to their original positions, and the circle remained stationary at the top of the second hill (helping event) or at the base of the first hill (hindering event). The length of the videos in each familiarization trial was 10 seconds, and the videos were repeated until each trial ended (S1 and S2 Videos).

The infants in the failed-helping condition underwent the same familiarization trials as those in the successful-helping condition, with two exceptions (see Fig 1). First, the helper started to push the circle toward the top of the second hill, but the two agents ended up slipping down to the bottom of the first hill together, where they remained stationary. Second, the hinderer pushed the circle down to the bottom and the two reached the base of the first hill together and remained stationary there instead of the hinderer going back to its original position. The purpose of the second change was to make the last scene of the helping and hindering events in this condition identical (S3 and S4 Videos).

**Fig 2:**
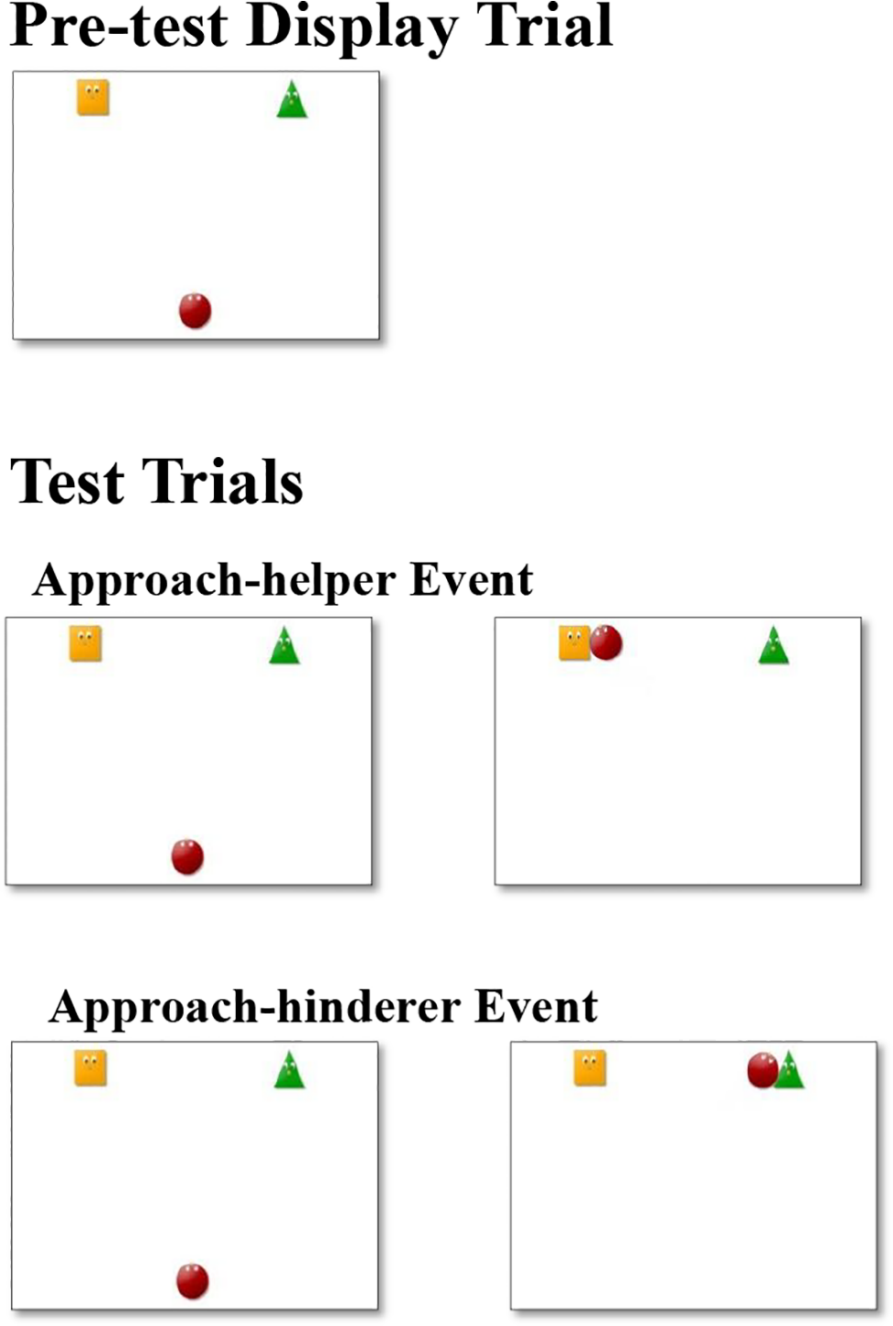
Example of the pre-test display trial and test trials for infants who saw the square help the circle and the triangle hinder the circle during the familiarization trials.

After the familiarization trials, the infants in both conditions were presented with a static pre-test display trial. The hills were absent, and only the three agents were present in the scene. The circle was centered at the bottom of the screen, and the square and the triangle were in either the upper left or upper right of the screen (see Fig 2). For 5 infants (3 infants in the successful-helping condition and 2 infants in the failed-helping condition), there are no looking-time data for the pre-test display trial, as they participated in a pilot experiment that had no pre-test display trial. Therefore, they were not included in the analyses of the pre-test display trial.

Finally, the infants in both conditions underwent a test trial (see Fig 2). For the first 4 seconds, the circle approached the agent who had helped it climb the hill (approach-helper event) or the agent who had hindered it from climbing the hill (approach-hinderer event). Then there was a 2-second pause. The video was repeated until the trial ended (S5 and S6 Videos).

The order of the familiarization events was counterbalanced. Sixteen infants saw the helping events during the first three familiarization trials and the hindering events during the last three familiarization trials. The other 16 infants saw the helping and hindering events in reverse order. The identities of the helper/hinderer and the locations (left or right) of the square and the triangle in the test videos were also counterbalanced.

The infants’ looking behavior was recorded by two observers. When the infant looked at the monitor, the observers pressed the button connected to a computer while viewing the infant through peepholes in the cloth-covered frames on either side of the monitor. The looking times recorded by the primary observer were used to determine the length of the trial.

In the familiarization phase, each trial ended when the infant looked away for 2 consecutive seconds, after having looked for at least 10 cumulative seconds, or when they looked for 60 cumulative seconds. In the pre-test display trial, each trial ended when the infant looked away for 2 consecutive seconds after having looked for at least 3 cumulative seconds, or when they looked for 35 cumulative seconds. In the test phase, each trial ended when the infant looked away for 2 consecutive seconds, after having looked for at least 4 cumulative seconds, or when they looked for 60 cumulative seconds. The observers’ agreement was measured for 27 out of 32 infants and averaged 92% per trial per infant.

### Results and Discussion

The preliminary analyses showed no significant interaction involving gender, the order of the familiarization events, the identities of the helper/hinderer, or the locations of the agents in the test trial, all *Fs*(1, 24) < 2.24, *p*s > .14. Therefore, the data were collapsed across these factors in subsequent analyses.

The infants’ looking times during the familiarization trials were averaged and analyzed by means of a 2 X 2 analysis of variance (ANOVA) with the condition (successful-helping or failed-helping) and test event (approach-helper or approach-hinderer) as between-subjects factors. The main effects of the condition, *F*(1, 28) = 3.99, *p* = .06, the test event, *F*(1, 28) < 1, and the interaction effect, *F*(1, 28) < 1, were nonsignificant, suggesting that the infants in the four groups tended to look for the same length of time during the familiarization trials (Successful-helping/approach-helper, *M* = 29.92, *SD* = 11.93; Successful-helping/approach-hinderer, *M* = 29.66, *SD* = 10.89; Failed-helping/approach-helper, *M* = 23.22, *SD* = 3.85; Failed-helping/approach-hinderer, *M* = 23.85, *SD* = 6.20).

The infants’ looking times during the pre-test display trial were analyzed as described above. The main effect of the test event, *F*(1, 23) < 1, and the interaction effect, *F*(1, 23) < 1, were nonsignificant. However, the main effect of the condition, *F*(1, 23) = 6.17, *p* = .02, was significant, suggesting that the infants in the successful-helping condition (*M* = 12.00, *SD* = 7.12) tended to look longer than those in the failed-helping condition (*M* = 6.82, *SD* = 2.20) during the pre-test display trial.

**Fig 3:**
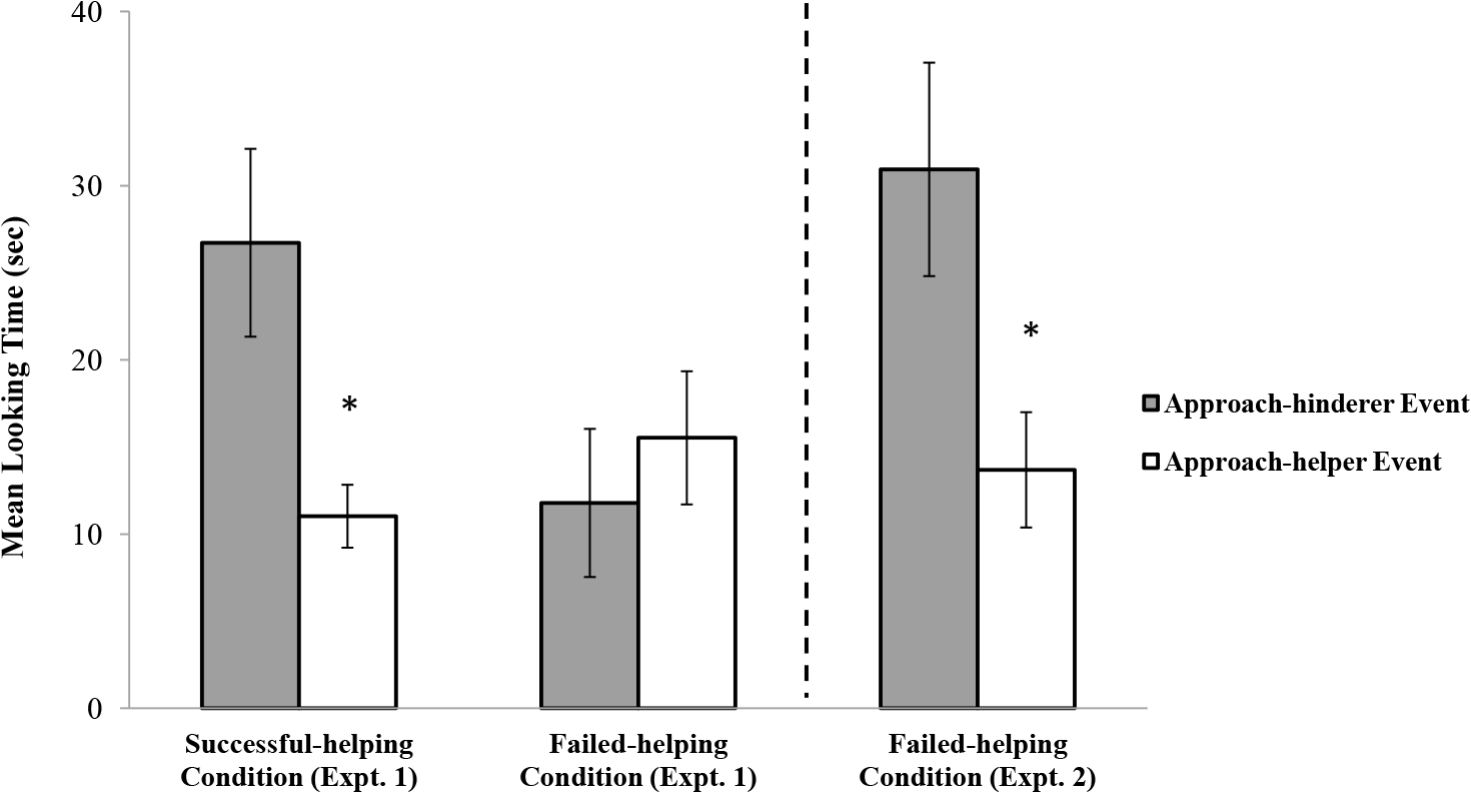
Infants’ mean looking times for the approach-helper and approach-hinderer events in Experiments 1 and 2.

The infants’ looking times during the test trial (see Fig 3) were analyzed as above. The main effects of the condition, *F*(1, 28) = 1.68, *p* = .21, and the test event, *F*(1, 28) = 2.20, *p* = .15, were nonsignificant. However, the interaction between the condition and the test event was significant, *F*(1, 28) = 5.81, *p* = .02, η_p_^2^ = .172. Planned comparisons indicated that (1) in the successful-helping condition, the infants who saw the approach-hinderer event (*M* = 26.73, *SD* = 15.26) looked for significantly longer than those who saw the approach-helper event (*M* = 11.03, *SD* = 5.11), *F*(1, 28) = 7.58, *p =* .01, η_p_^2^ = .213, and (2) in the failed-helping condition, there was no difference in the looking times between the infants who saw the approach-hinderer event (*M* = 11.79, *SD* = 12.03) and those who saw the approach-helper event (*M* = 15.53, *SD* = 10.81), *F*(1, 28) < 1. Non-parametric Wilcoxon rank-sum tests confirmed the results of the successful-helping (*W*s = 44, *p* = .01) and failed-helping (*W*s = 50, *p* = .07) conditions. The rank sums for the approach-hinderer and the approach-helper test event groups were 92 and 44, respectively, in the successful-helping condition, and were 50 and 86, respectively, in the failed-helping condition. Post hoc G-power analyses with α at .05 (two-tailed) [9] indicated a 73% chance of detecting the effect size observed in the successful-helping condition (*d* = 1.38; a large effect according to Cohen’s effect size conventions [10]) and a 9% chance of detecting the effect size observed in the failed-helping condition (*d* = .33; a small effect [10]).

The results of an analysis of covariance (ANCOVA), with the infants’ mean looking times during the familiarization and pre-test display trials as a covariate, replicated those of the ANOVA. The interaction between the experimental condition and the test event was again significant, *F*(1, 27) = 7.55, *p* = .01, η_p_^2^ = .219.

As in previous research [2–4], the 12-month-olds showed a significant difference in the length of time they looked at the approach-hinderer event vs. the approach-helper event in the successful-helping condition. Thus, when the square’s and triangle’s actions yielded the intended outcome, the 12-month-old infants were able to infer the circle’s preference toward one of them and expected the circle to approach the one that had previously helped it. However, in the failed-helping condition, the 12-month-old infants did not show a reliable difference in the looking times for the approach-hinderer and the approach-helper events. The results suggest that 12-month-olds have difficulty attributing social preferences to others on the basis of intention when the helper intends, but fails, to successfully help the agent.

One might argue that the failed-helping video might not have clearly portrayed the helper’s intention to help the circle. To examine this possibility, 10 adults (5 male, 5 female; mean age = 24.1 years) were recruited to participate in the study and were presented with the failed-helping video. Five adults (2 male, 3 female) were randomly assigned to a video in which the square was the failed-helper. The other 5 adults (3 male, 2 female) were shown a movie in which the triangle was the failed-helper. A researcher told the participants, “You are going to watch a 20-second long video. Afterwards, you will be asked to write about what you saw in the video.” The participants were shown the 10-second long failed-helping event twice on a laptop. After the participants watched the event, they were asked to write one or two sentences recounting what they had seen in the video.

The written descriptions suggested that all participants properly analyzed the behaviors in the failed-helping event. For example, they all identified either the square or the triangle as a helper by using the word “helper” or “help” in their descriptions. Also, 8 out of the 10 participants showed an understanding that the two agents had failed to reach the top of the hill. For instance, they specifically used the words “failed,” “did not succeed,” “fell to the base,” or “unable to reach the top.” The other 2 out of the 10 participants did not mention the failure and only described the helping behaviors. Thus, the failed-helping video was readily interpreted by adults as we intended.

In Experiment 2, we investigated whether older infants, i.e., 16-month-olds, are capable of inferring that the circle would prefer a helpful agent to a hindering agent, even when the helpful agent failed to help the circle to achieve its goal. We predicted that 16-month-old infants should be able to do so because infants at this age showed an understanding of intentional actions in prior studies (e.g., [11, 12]).

## Experiment 2

### Methods

#### Participants

Sixteen healthy, full-term infants participated in the study (7 female, mean age = 15 months and 24 days, age range = 14 months and 28 days to 17 months and 10 days). Another 13 infants were tested but not included in the analyses because of fussiness (4), inattentiveness (2), drowsiness (1), inappropriate postures (turning their backs to the monitor) during the test trial (2), parental interference (2), or observers' difficulty with seeing infants’ eyes (2). Half of the infants observed the approach-helper event and the other half observed the approach-hinderer event during the test trial. Each infant’s parent gave written informed consent, and the protocol was approved by the Institutional Review Board at Yonsei University.

#### Stimuli and procedure

The stimuli and procedure of Experiment 2 were identical to those of the failed-helping condition in Experiment 1, with the following exceptions: first, the infants underwent 4, instead of 6, familiarization trials, consisting of two helping and two hindering trials. Second, there was no pre-test display trial in Experiment 2. These changes were introduced to reduce the likelihood that the 16-month-olds would get bored during the trials.

Nine of the infants saw the helping events during the first two familiarization trials and the hindering events during the last two familiarization trials. The other 7 saw the helping and hindering events in reverse order. The identities of the helper/hinderer and the locations (left or right) of the square and the triangle in the test videos were roughly counterbalanced. The observers’ agreement was measured for all of the infants and averaged 95% per trial per infant.

### Results and Discussion

The preliminary analyses showed no significant interaction involving gender, the order of the familiarization events, the identities of the helper/hinderer or the locations of the agents, all *F*s(1, 12) < 1.36, *p*s > .26. Therefore, the data were collapsed across these factors in subsequent analyses.

The infants’ looking times during the familiarization trials were averaged and analyzed by means of a one-way ANOVA with the test event (approach-helper or approach-hinderer) as a between-subjects factor. The effect of the test event on the infants’ looking times during the familiarization trials was not significant, *F*(1, 14) < 1, suggesting that the infants who saw the approach-helper event and those who saw the approach-hinderer event tended to look for the same amount of time during the familiarization trials (approach-helper event, *M* = 34.69, *SD* = 11.33; approach-hinderer event, *M* = 36.41, *SD* = 9.28).

The infants’ looking times during the test trial (see Fig 3) were analyzed by means of a one-way ANOVA with the test event (approach-helper or approach-hinderer) as a between-subjects factor. The effect of the test event was significant, *F*(1, 14) = 6.13, *p* = .03, η^2^ = .305, suggesting that the infants looked for longer at the approach-hinderer event (*M* = 30.94, *SD* = 17.33) than at the approach-helper event (*M* = 13.69, *SD* = 9.36). Non-parametric Wilcoxon rank-sum tests confirmed the difference in the looking times between the approach-helper event and the approach-hinderer event, *W*s = 46, *p* = .02. In Experiment 2, the rank sums for the approach-hinderer and the approach-helper events were 90 and 46, respectively. Post hoc G-power analysis (α at .05, two-tailed) [9] indicated a 63% chance of detecting the effect size (*d* = 1.24; a large effect [10]).

An ANCOVA showed that the same test results were obtained after adjusting for the differences in the infants’ mean looking times during the familiarization trials. The analysis yielded a significant main effect of the test event, *F*(1, 13) = 5.65, *p* = .03, η_p_^2^ = .303.

Next, we compared the 12-month-olds’ looking times in the failed-helping condition of Experiment 1 with those of the 16-month-old infants in Experiment 2. The infants’ looking times during the test trial were analyzed by means of a 2 X 2 ANOVA with age (12-months-old or 16-months-old) and test event (approach-helper or approach-hinderer) as between-subjects factors. The main effects of the test event, *F*(1, 28) = 2.25, *p* = .15, and age, *F*(1, 28) = 3.69, *p* = .07, were nonsignificant. However, the interaction between the age and the test event was significant, *F*(1, 28) = 5.42, *p* = .03, η_p_^2^ = .162. Planned comparisons indicated that (1) the 16-month-old infants who saw the approach-hinderer event looked for significantly longer than those who saw the approach-helper event, *F*(1, 28) = 7.33, *p* = .01, η_p_^2^ = .207, but (2) the 12-month-olds looked for nearly an equal amount of time at the approach-hinderer event and at the approach-helper event, *F*(1, 28) < 1.

Unlike the 12-month-olds in Experiment 1, the 16-month-old infants looked for a significantly longer period at the approach-hinderer event than at the approach-helper event. These results tell us that the 16-month-old infants (1) differentiated the helper from the hinderer, despite the fact that the helper did not successfully help the agent reach the top of the hill, (2) expected that the agent would approach the helper and (3) were surprised when they saw the agent approaching the hinderer. These results indicate that 16-month-old infants can infer the social preference of an agent on the basis of intention.

Then, why were the 12-month-old infants in Experiment 1 unable to attribute a disposition to the circle when the helping agent failed to help the circle achieve its goal? There are at least three possible explanations for this discrepancy. One is that 12-month-old infants may simply interpret the circle’s approach actions during the test at a purely behavioral level. They may have expected the circle to move toward the agent that is positively associated with goal achievement rather than toward the agent that is not positively associated with goal achievement. This explanation is consistent with the view that the infants simply interpret others’ actions in terms of the most efficient means to achieve a goal [13].

Another possibility is that 12-month-olds may have simply relied on perceptual associations between the agents. In particular, 12-month-olds in the failed-helping condition may have formed an equal association between the circle and the other agents, because either the helper or the hinderer was right next to the circle in the final scenes. The 12-month-old infants in the condition might have simply attended to the equal association between the agents, which might have hindered them from detecting the circle’s disposition toward the helper.

The third possibility is that 12-month-olds looked equally in the failed-helping condition, not because they cannot consider intention information, but because their limited information-processing abilities may have prevented them from understanding a failed-helping situation in which a conflict between intention and outcome occurs (i.e., the helper’s intention was positive but its outcome was negative).

In Experiment 3, we examined these possibilities in 12-month-olds by reducing the cognitive processing load. The infants did not witness the action outcome, so the conflict between the intention and the outcome was eliminated. If the first and second explanations were correct, we predicted that infants would look for about equal lengths of time at the two test events, because (1) neither helping nor hindering events were associated with the circle’s goal achievement, and (2) the events ended with the circle next to either the helper or hinderer, which would result in equal association between the agents, as in the failed-helping condition. However, if the third explanation were true, the infants would more readily expect the circle to approach the agent who intended to help it. Therefore, they would look for significantly longer at the approach-hinderer event than at the approach-helper event.

## Experiment 3

### Methods

#### Participants

Sixteen healthy, full-term infants participated in the study (8 female, mean age = 12 months and 12 days, age range = 11 months and 13 days to 13 months and 14 days). Another 5 infants were tested but not included in the analyses because of fussiness (2), inattentiveness (2), or observers' difficulty with seeing infants’ eyes (1). Eight infants observed the approach-helper event and the other 8 infants observed the approach-hinderer event. Each infant’s parent gave written informed consent, and the protocol was approved by the Institutional Review Board at Yonsei University.

#### Stimuli and procedure

**Fig 4:**
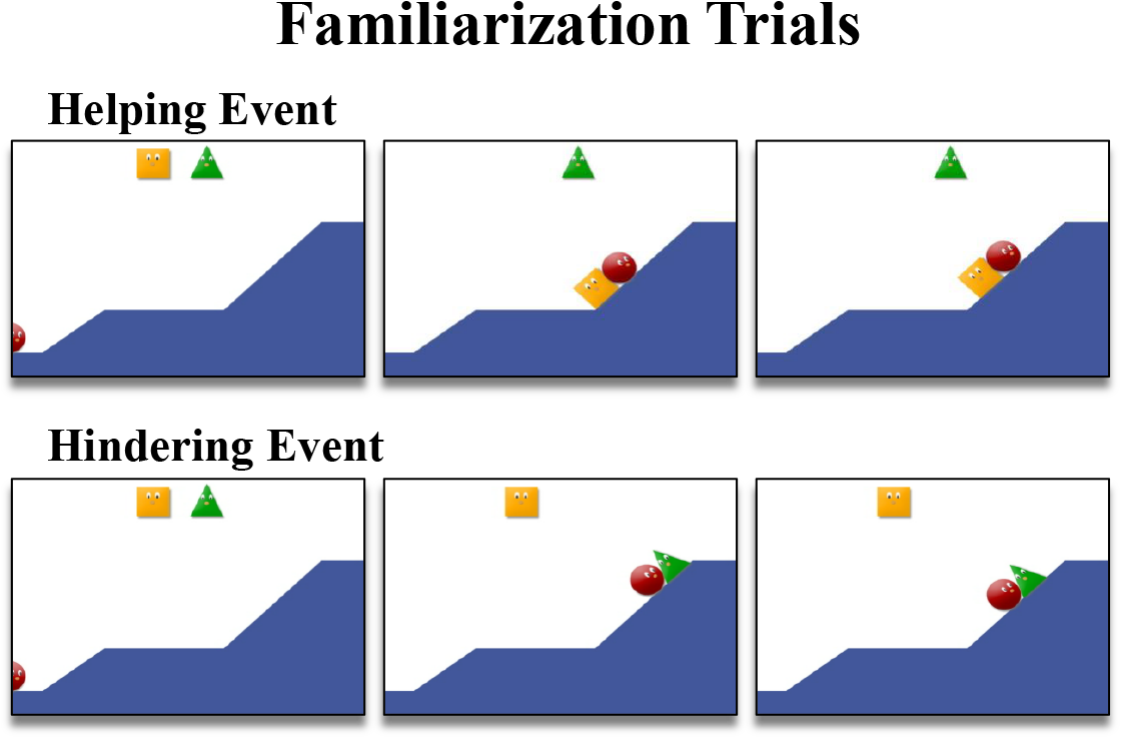
Example of the familiarization trials in Experiment 3.

The stimuli were similar to those used in Experiments 1 and 2 except for the final positions of the circle during familiarization events: Both helping and hindering events ended when the circle was at the middle of the hill (see Fig 4). In the helping event, the circle reached the top of the first hill and then attempted to reach the top of the second hill but slid back to the base of the second hill. The circle was pushed upward by the helper (e.g., a square; helping event) and the circle reached the middle of the second hill and the two agents remained stationary there. In the hindering event, the circle reached the top of the first hill and then attempted to reach the top of the second hill but was pushed downward by the hinderer (e.g., a triangle; hindering event). The circle slid back to middle of the second hill and the two agents remained stationary there. Thus, final locations of the circle in both helping and the hindering events were identical and it was ambiguous as to whether the circle would eventually succeed or fail in reaching the top of the hill. The familiarization video consisted of this 7-second animated scene, a 1-second paused final scene and a 1-second black screen. The videos were repeated until each trial ended (S7 and S8 Videos).

The order of the familiarization events and the identities of the helper/hinderer were counterbalanced. The locations (left or right) of the square and the triangle in the test videos were roughly counterbalanced.

As in Experiment 1, infants underwent 6 familiarization trials, 1 pre-test display trial, and 1 test trial. The videos were accompanied by an excerpt from Wolfgang Amadeus Mozart's Piano Sonata No. 11 in A major, KV 331, in order to draw the infants’ attention toward the videos. In the familiarization phase, each trial ended when the infant looked away for 2 consecutive seconds after having looked for at least 7 cumulative seconds, or when they looked for 54 cumulative seconds. In the pre-test display trial, each trial ended when the infant looked away for 2 consecutive seconds after having looked for at least 3 cumulative seconds, or when they looked for 35 cumulative seconds. In the test phase, each trial ended when the infant looked away for 2 consecutive seconds after having looked for at least 4 cumulative seconds, or when they looked for 60 cumulative seconds. The observers’ agreement was measured for 13 out of 16 infants and averaged 93% per trial per infant.

### Results and Discussion

The preliminary analyses showed no significant interaction involving gender, the order of the familiarization events, the identities of the helper/hinderer, or the locations of the agents, all *F*s(1,12) < 2.08, *p*s > .17. Therefore, the data were collapsed across these factors in subsequent analyses.

The infants’ looking times during the familiarization trials were averaged and analyzed by means of a one-way ANOVA with the test event (approach-helper or approach-hinderer) as a between-subjects factor. The effect of the test event on the infants’ looking times during the familiarization trials was not significant, *F*(1, 14) < 1, suggesting that the infants who saw the approach-helper event and those who saw the approach-hinderer event tended to look for the same amount of time during the familiarization trials (approach-helper event, *M* = 28.36, *SD* = 5.83; approach-hinderer event, *M* = 25.43, *SD* = 6.37).

Analysis of the infants’ looking times during the pre-test display trial produced similar results. The effect of the test event on the infants’ looking times during the pre-test display trial was not significant, *F*(1, 14) < 1, suggesting that the infants who saw the approach-helper event and those who saw the approach-hinderer event tended to look for the same amount of time during the pre-test display trial (approach-helper event, *M* = 10.85, *SD* = 9.87; approach-hinderer event, *M* = 13.14, *SD* = 10.68).

**Fig 5:**
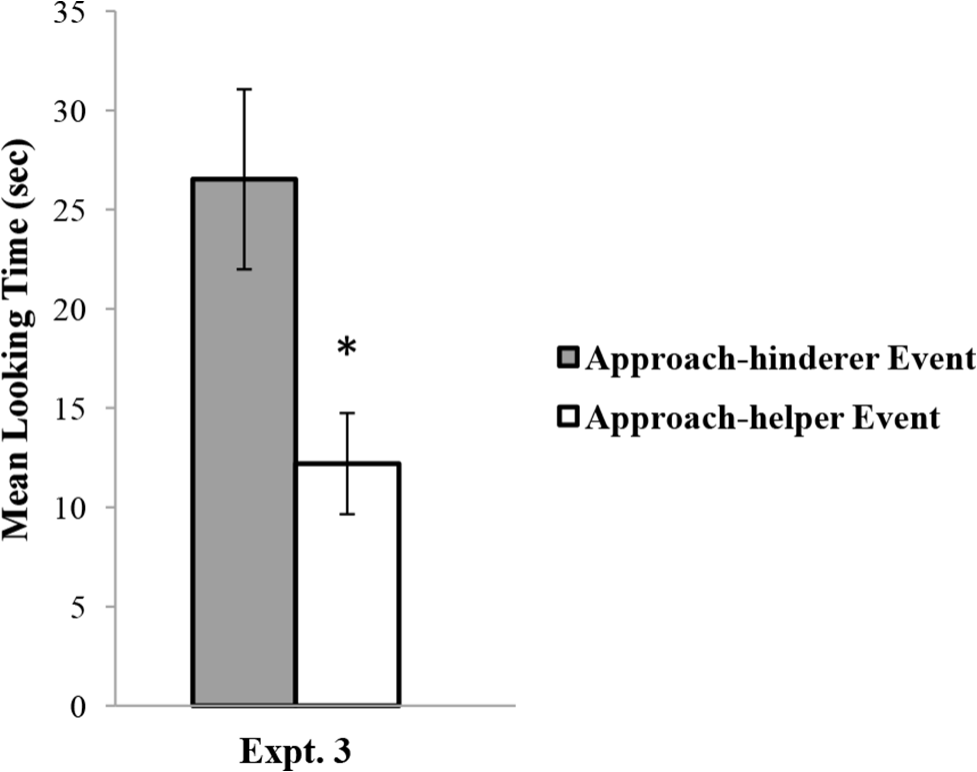
Infants’ mean looking times for the approach-helper and approach-hinderer events in Experiment 3.

The infants’ looking times during the test trial (see Fig 5) were analyzed by means of a one-way ANOVA with the test event (approach-helper or approach-hinderer) as a between-subjects factor. The effect of the test event was significant, *F*(1, 14) = 7.59, *p* = .02, η^2^ = .351, suggesting that the infants looked longer at the approach-hinderer event (*M* = 26.53, *SD* = 12.82) than at the approach-helper event (*M* = 12.20, *SD* = 7.20). Non-parametric Wilcoxon rank-sum tests confirmed the difference in the looking times between the approach-helper event and the approach-hinderer event, *W*s = 48, *p* = .04. In Experiment 3, the rank sums for the approach-hinderer and the approach-helper events were 88 and 48, respectively. Post hoc G-power analysis (α at .05, two-tailed) [9] indicated a 73% chance of detecting the effect size (*d* = 1.38; a large effect [10]).

The results of the ANCOVA with the infants’ mean looking times during the familiarization and pre-test display trials as a covariate replicated those of the ANOVA. The analysis yielded a significant main effect of the test event, *F*(1, 13) = 12.66, *p* = .004, η_p_^2^ = .493.

When the infants did not witness the final outcomes of helping and hindering actions, 12-month-olds performed similarly to the 16-month-olds in Experiment 2: they looked significantly longer at the approach-hinderer event than at the approach-helper event. The results allowed us to rule out the possibilities (1) that 12-month-old infants in Experiment 1 merely expected the circle to approach the agent that was associated with positive outcomes during the test and (2) that they relied on perceptual associations between the agents. Instead, the results suggest that 12-month-olds might have had difficulties in attributing social preferences to others on the basis of intentions in the failed-helping condition of Experiment 1 due to the cognitive load required for processing events in which the outcome and the intention conflicted. When the conflict between the intention and the outcome was eliminated, 12-month-old infants were able to take intentions into account when reasoning about third party’s social preference.

## General Discussion

The present research expands on previous studies [1, 2, 4] by demonstrating that infants understand that others prefer a helpful agent. The previous studies did not evince whether infants based their attribution of others’ disposition on the intention or the outcome of prior helping actions. The current research revealed that infants as young as 12-months of age infer an agent’s disposition using intentionality information. Thus, the ability to infer a third party’s social preference based on prior interaction is present in infants from as young as 10-months-old [2], but the ability to focus on the intentions behind the previous actions when inferring third party’s social preference emerges by 12-months of age.

The current findings add to the emerging evidence on moral sensitivity in infancy. When the intention and outcome of helping behaviors are consistent, even 3-month-old infants [14, 15] discriminate helpers from hinderers. However, when the intention and outcome of the helping behaviors are divergent, infants are able to generate their own preference for a helpful agent from 8-months of age [5]. Our findings revealed that 16 month-olds, but not 12-month-olds, infer the circle’s preferences toward another agent based on intentionality information in prior interactions when the intention and the outcome were pitted against each other. However, when they did not witness event outcomes and, thus, there was less information to process, 12-month-old infants were able to infer the circle’s disposition by using information regarding how another agent intended to help the circle in previous familiarization events.

Note that across the three experiments, infants’ looking times at the approach-helper event are similarly short, suggesting the possibility that even 12-month-olds in the failed-helping condition of Experiment 1 might have detected the failed-helper’s intention. In contrast, all infants looked longer to the approach-hinderer event, with the exception of the 12-month-olds in the failed-helping condition of Experiment 1. So, we could speculate that 12-month-olds in the failed-helping condition might have expected the circle to approach the helper, but the identical behavioral outcomes between the failed-helping and the hindering events might have prevented them from generating an expectation about how the circle would act toward the hinderer. This is one possible reason why 12-month-olds in the failed-helping condition looked with an equally short duration at the approach-helper and approach-hinderer events. This possibility should be explored in the future research.

Taken together, the current results consistently suggest that the 12-month-olds in the failed-helping condition of Experiment 1 may have failed in inferring others’ dispositions due to their limited information-processing capacities, not because of either their action analysis at a simple behavioral level or mere associations between the agents. We suggest there are at least two possible ways in which the information processing mechanism guiding moral development develops during infancy. The first possibility is that the difference between younger and older infants may be due to a delay in the maturation of a reasoning mechanism that is specific to the moral domain. For instance, there might be a shift from outcome-based to intention-based moral reasoning process within a moral domain (see [16] for a similar idea on preschoolers’ moral development). Traditional findings, using explicit, verbal responses, have suggested that young children’s moral judgment relies mainly on outcome information and later becomes sensitive to intentionality over the course of development [8]. However, some recent findings have refuted this possibility. In Hamlin [5], 5- and 8-month-olds did not evaluate helping and hindering agents based solely on the outcomes; for example, they did not differentiate between the successful and failed helpers. Consistently, the current research revealed that both 12- and 16-month-olds possess some sensitivity to intentions. Thus, there is no clear evidence that outcome-based moral evaluation is fully replaced by intention-based moral evaluation during infancy. Rather, it is possible that both evaluation processes develop simultaneously from a certain point of development [16].

The other possibility is that younger and older infants may share the same built-in moral reasoning mechanism, but only older infants can make use of it due to their more efficient domain-general information processing abilities (see [17] for a review). The 12-month-olds in the failed-helping condition of Experiment 1 may have been unable to infer others’ dispositions due to their relatively limited information-processing abilities outside the moral domain, such as executive function, preventing the expression of infants’ psychological understanding [18]. It has been suggested that 12-month-olds have some ability to represent the internal states of the recipient of helping or hindering actions [2, 4] and that they are able to process conflicting information between the outcome of an action and the intention behind it [5]. However, due to their limitations in terms of domain-general processing abilities, the 12-month-olds in this study might have had difficulty with both representing the mental states of the agent and processing the intention/outcome conflict simultaneously. The results from Experiment 3 are consistent with this possibility because 12-month-olds successfully use intention information to attribute a disposition to another agent, when the cognitive load is reduced.

Infants can detect the intentions underlying others’ goal-directed actions even during the first year of their life [19, 20], and they may possess early biases to focus on intentionality in their moral understanding. Intentions, not outcomes, may be more reliable cues to use when predicting who will be helpful in the future; thus, a tendency to pay attention to intentions may be naturally selected [5]. This is consistent with the view of human moral sense as a built-in, innate mechanism that has its origin in evolution [21, 22].

It is also possible that early social experiences influence infants’ early emerging moral sensitivity. Infants in their first year of life engage in limited social interactions with their peers [23]. Between 1 and 2 years of age, infants begin to view other infants as playmates and engage in coordinated interactions, such as offering an object, sharing objects, and mutual imitations [24–26]. Thus, social interactions and experiences with parents and peers may contribute to the emergence of moral understanding in infants.

Infants’ social experiences with failed helping actions may be especially critical for their ability to infer a third party’s social preference based on helpful intentions. Although they readily display their own preference for a person who intends, but fails, to be helpful [5], it may not be easy for young infants to infer how others would react to failed helpers without having had specific opportunities to observe or interact with others. Infants could learn that even failed helping actions may be viewed as nice by interacting with their parents. For instance, parents would praise their infant for being helpful even when the infant drops a requested toy while handing it to the parents. Through such social experiences, infants could learn that others’ social preferences, like their own, are sometimes determined by intentionality information, and therefore become increasingly competent in morally relevant cognitions and evaluations [27]. Future research examining much younger infants is required to explore the contribution of inborn social biases and varied social experiences to the developmental origin of intention-based moral sensitivity.

## Supporting Information

S1 Video Example of a helping event in the successful-helping condition. (AVI)

S2 Video Example of a hindering event in the successful-helping condition. (AVI)

S3 Video Example of a helping event in the failed-helping condition. (AVI)

S4 Video Example of a hindering event in the failed-helping condition. (AVI)

S5 Video Example of an approach-helper event for infants who saw the square help the circle and the triangle hinder the circle during the familiarization trials. (AVI)

S6 Video Example of an approach-hinderer event for infants who saw the square help the circle and the triangle hinder the circle during the familiarization trials. (AVI)

S7 Video Example of a helping event in Experiment 3. (AVI)

S8 Video Example of a hindering event in Experiment 3. (AVI)
